# Cloning, optimization of induction conditions and purification of *Mycobacterium tuberculosis* Rv1733c protein expressed in *Escherichia coli*

**Published:** 2017-04

**Authors:** Mitra Ashayeri-Panah, Fereshteh Eftekhar, Bahram Kazemi, Joan Joseph

**Affiliations:** 1 Department of Microbiology, Faculty of Biological Sciences and Technology, Shahid Beheshti University, Tehran, Iran; 2 Cellular and Molecular Biology Research Center, Shahid Beheshti University of Medical Sciences, Tehran, Iran; 3 Hospital Clinic/HIVACAT, School of Medicine, University of Barcelona, Barcelona, Spain

**Keywords:** *Mycobacterium tuberculosis*, Rv1733c, Cloning, Expression optimization, Protein purification

## Abstract

**Background and Objectives:**

Rv1733c is a latency antigen from *Mycobacterium tuberculosis*, a probable integral-membrane protein with promiscuous T-cell and B-cell epitopes, making it a potential vaccine candidate against tuberculosis. This study aimed to clone and optimize the expression of recombinant Rv1733c in *Escherichia coli* for purification.

**Materials and Methods:**

Chemically synthesized *rv1733c* coding sequence was cloned in pET-23a(+) followed by transforming *E. coli* BL21 (DE3) cells. To evaluate the induction conditions for optimized expression, factorial design of experiments was employed using four different media as well as four levels of isopropyl-b-D-thiogalactopyranosid [IPTG] concentration and duration of induction. The recombinant protein was then purified using a His-tag purification kit and detected through western blotting.

**Results:**

Recombinant Rv1733c (> 24 kDa) was expressed and accumulated in the cytoplasm of the *E. coli* cells. Medium composition showed the most significant effect on the yield of the recombinant protein (P = 0.000). The highest yield of recombinant Rv1733c occurred in the presence of 0.4 mM of IPTG in Terrific Broth medium (containing 1.2% tryptone, 2.4% yeast extract, 72 mM K _2_ HPO _4_ , 17 mM KH _2_ PO _4_ and 0.4% glycerol) after 10 h at 37°C. Under these conditions, the expression level was around 0.5 g/L of culture medium. Purified Rv1733c was detected by an anti-polyhistidine antibody and a tuberculosis patient’s serum. Systematic optimization of induction conditions gave us high yield of recombinant polyhistidine-tagged Rv1733c in *E. coli* which was successfuly purified.

**Conclusion:**

We believe that the purified Rv1733c recombinant protein from *M. tuberculosis* might be a good candidate for vaccine production against tuberculosis.

## INTRODUCTION

*Mycobacterium tuberculosis*, the main cause of tuberculosis in humans, remains a serious global health threat. It is estimated that approximately 2–3 billion people are already infected with *M. tuberculosis*, making eradication of tuberculosis difficult (1). Immunization with *Mycobacterium bovis* Bacillus Calmette-Guerin (BCG), the only available vaccine against tuberculosis, fails to induce adequate immune responses to latency-associated antigens of *M. tuberculosis* (2, 3). Thus, choosing a candidate antigen out of these and employing the relevant recombinant protein in a BCG prime/protein boost regimen might improve upon BCG vaccination alone (4).

**Fig. 1.**
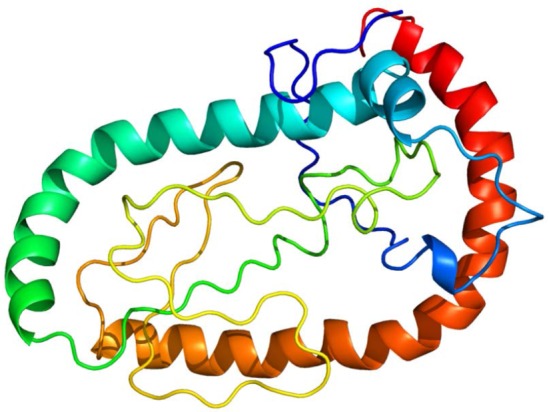
A three-dimensional structural model of Rv1733c created using Phyre2. The model was predicted using the structure of hydrolyzed trypsin inhibitor v from *Cucurbita maxima* (Protein Data Bank code 1HYN) as the template with a confidence of 61.6 and a percent I.D. of 44.

Rv1733c is a latency antigen of *M. tuberculosis* predicted to be a conserved transmembrane protein with two transmembrane helices (5). The three-dimensional structure of Rv1733c as predicted by the Protein Homology/Analogy Recognition Engine (PHYRE) version 2.0, shows low level of complexity with two long α-helices and no β-sheet (Fig. 1) (6). Rv1733c is a probable non-allergenic antigen containing numerous B-cell epitopes and multiple human histocompatibility leukocyte antigen (HLA) class I and HLA class II epitopes (7–11). Such information from bioinformatics databases suggest Rv1733c as a pertinent candidate for developing vaccines against tuberculosis. Therefore, production of recombinant Rv1733c may be worthwile for testing its potential as a vaccine candidate.

Expression of recombinant proteins in *E. coli* is a preferred inexpensive and easy method for large-scale production of many proteins (12). However, expression of transmembrane proteins is challenging due to low expression levels, high tendency to aggregate in inclusion bodies, cellular toxicity and plasmid instability (13). In such cases, expression has to be optimized thoughtfully to minimize the efforts in subsequent purification steps. Such optimization procedures are mainly based on decreasing culture volume and increasing the yield of soluble target proteins (14).

Induction conditions such as type of culture media, concentration of inducer, post-induction temperature and time have been shown to influence protein expression in *E. coli* (15). These factors have usually been optimized one at a time. However, statistical designed experiments which consider interactions between variables, are gaining success in optimizing the production of recombinant proteins (15). Also, the composition of the lysis buffer is an important factor which affects obtaining soluble recombinant proteins and if possible, omitting the use of detergents which may interfere with the down-stream purification process (15, 16).

The aim of this study was to clone and express Rv1733c protein in *E. coli* and optimize the expression of the recombinant protein under different induction conditions designed by factorial method to obtain high yield of the protein and finally purify it.

## MATERIAS AND METHODS

### Molecular cloning and construction of the expression plasmid.

The *rv1733c* sequence derived from *M. tuberculosis* H37Rv (accession number JLDD01000018.1) was chemically synthesized (Shingene, China) and was provided as PCR product. *Bam*HI and *Hin*dIII restriction sites were incorporated in the sequence to allow in-frame cloning of the PCR product into the corresponding restriction sites in the polylinkers of pET23(a)+ plasmid (Novagen, Madison, USA). The gene was cloned without the stop codon in order to allow incorporation of the polyhistidine (His) tag of pET23a to the C-terminus of the protein. The vector pET23a and the PCR product were digested with the corresponding restriction enzymes (NEB, Ipswich, MA) and ligated with T4 ligase (NEB). *E. coli* TOP10 (Invitrogen, Paisley, UK) was used as the host for the cloned construction. The recombinant plasmid was confirmed by agarose gel electrophoresis before and after *Bam*HI/*Hin*dIII digestion followed by sequencing analysis. The recombinant plasmid was then used to transform *E. coli* BL21 (DE3) (Invitrogen) (17).

### Small scale expression and solubility testing.

A single colony of the recombinant *E. coli* BL21 (DE3) was inoculated into 5 ml Luria-Bertani (LB) broth (Sigma-Aldrich, MO, USA) supplemented with 100 μg/ml ampicillin (Sigma-Aldrich) and grown overnight. The culture was diluted (1:10 ml) with fresh LB broth and incubated at 37°C for 2 h to obtain an optical density of 0.6 at 600 nm. One ml of this non-induced culture was saved as control and preliminary induction of expression was carried out by adding 0.3 and 0.4 mM isopropyl-b-D-thiogalactopyranosid (IPTG) (Sigma-Aldrich). The cultures were incubated at 37°C and 1 ml samples were taken every hour up to 8 h. The cells were separated by centrifugation (6000 g, 5 min) and the supernatant proteins were precipitated by 80% (w/v) ammonium sulfate (Sigma-Aldrich) (18). The cell pellets and the protein precipitates were resuspended in 50 μl phosphate-buffered saline (PBS, pH 7.4) (Sigma-Aldrich), mixed with 50 μl of 2X Laemmli Sample Buffer (Bio-Rad, CA, USA) with 5% (v/v) β-mercaptoethanol (Sigma-Aldrich) and were heated 10 min at 100°C. The resulting cell lysates were collected by centrifugation (13,000 g, 30 min, 4°C) and the supernatants were saved for solubility testing. Culture supernatant precipitates, total cell lysate, supernatants and pellets of cell lysates were further analysed by SDS-PAGE and western blot.

### Optimization of induction conditions.

To determine the optimum induction temperature, recombinant *E. coli* BL21 was grown at 37°C to OD _600_ of 0.6 before the addition of IPTG (0.4 mM). The cultures were incubated at 37°C, 30°C, and 25°C for up to 22 h. One ml samples were taken every hour from T1 to T8 at 37°C, from T8 to T15 at 30°C and T15 to T22 at 25°C and supernatants of cell lysates were analyzed by SDS-PAGE. Further optimization experiments were continued at 37°C. Using the factorial design of experiments, media composition, IPTG concentration and time after induction were varied. Four levels were tested for each variable and the formulation for the tested media were all from Cold Spring Harbor Protocols website at http://www.cshprotocols.cshlp.org/ with slight modifications (Tables 1 and 2). An orthogonal array design consisting of 16 experiments was also employed to confirm the result for each factor (Table 3). Cell growth at the final expression time at 37°C was measured by OD _600_ . Cells were harvested by centrifugation at 6000 g for 5 min and the pellets were stored at −20°C for further analysis by SDS–PAGE. The expression level at each condition was compared with the total protein extract from the un-induced sample control.

**Table 1.** Different levels tested for three variables of medium composition, inducer concentration and induction time based on factorial design.

**Medium Composition^1^**	**Induction Time (h) at 37°C**	**IPTG^2^ Concentration (mM)**
2x YT (2x Yeast extract and Tryptone) broth: 1.6% tryptone, 1% yeast extract, and 0.5% NaCl	7	0.1
TB (Terrific Broth) medium: 1.2% tryptone, 2.4% yeast extract, 72 mM K_2_HPO_4_, 17 mM KH_2_PO_4_ and 0.4% glycerol	8	0.2
SB (Super Broth) medium: 3% tryptone, 2% yeast extract, and 0.5% NaCl	9	0.3
LB (Luria-Bertani) broth with 0.5% NaCl: 1% tryptone, 0.5% yeast extract, and 0.5% NaCl	10	0.4

1 The media ingredients were all purchased from Merck, Darmstadt, Germany

2 isopropyl-b-D-thiogalactopyranosid

**Table 2.** Factorial design of experiments on the effect of medium composition, inducer concentration and induction time on the yield of recombinant Rv1733c.

**Factor**	**Level**	**No. of experiments**	**Mean intensity of the protein bands+ SD^2^**	**F^3^**
**Medium Composition**	2x YT broth	16	1110414300 ± 33498401	79.999^**^
	TB medium	16	2002756624 ± 50092673	
	SB medium	16	1164244815 ± 45048623	
	LB broth	16	1147343964 ± 60453014	
**IPTG^1^ Concentration**	0.1 mM	16	1296686639 ± 105969007	0.199^ns^
	0.2 mM	16	1354345977 ± 110538361	
	0.3 mM	16	1360018090 ± 100495267	
	0.4 mM	16	1413708997 ± 112120518	
**Induction Time**	7 h	16	1358243836 ± 86796572	0.075^ns^
	8 h	16	1394462190 ± 97065514	
	9 h	16	1348779185 ± 127775831	
	10 h	16	1323274493 ± 114544465	

1 isopropyl-b-D-thiogalactopyranosid

2 standard deviation

3 The significance level was determined using ANOVA,

* 5% level,

** 0.1% level and ns non-significant.

**Table 3.** Orthogonal array design consisted of 16 experiments.

**Experiment No.**	**Medium Composition^1^**	**IPTG^2^ Concentration (mM)**	**Induction Time (h)**	**Score^3^**
1	LB	0.2	8	16
2	LB	0.1	7	5
3	YT	0.4	9	12
4	LB	0.3	9	10
5	SB	0.3	8	6
6	LB	0.4	10	9
7	SB	0.1	10	13
8	TB	0.4	8	2
9	TB	0.3	7	4
10	SB	0.4	7	8
11	TB	0.2	10	3
12	YT	0.2	7	7
13	YT	0.3	10	14
14	YT	0.1	8	11
15	SB	0.2	9	15
16	TB	0.1	9	1

1 YT: 2x Yeast extract and Tryptone broth; TB: Terrific Broth medium; SB: Super Broth medium; LB: Luria-Bertani broth with 0.5% NaCl

2 isopropyl-b-D-thiogalactopyranosid

3 Scores were determined between 1 (the worst) and 16 (the best) based on the quantification of recombinant Rv1733c bands in SDS–PAGE gel using ImageQuant TL software.

### Lysis buffer selection.

Five different lysis buffers were tested as shown in Table 4 and the buffer with the highest yield of soluble Rv1733c was selected for further experiments. Two ml of each buffer was added to cell pellet from 5 ml induced culture and the lysis was carried out according to the reference articles with slight modifications. For buffers 1 to 3 and buffer 5 sonication 6 x 20 s was employed using a Branson Sonifier equipped with a microtip (Branson Sonifier 250, Branson Ultrasonic, Danbury, CT, USA).

**Table 4.** Different lysis buffers composition.

**No.**	**Buffer Ingredients**	**Final Concentration**	**Reference**
1	Tris-HCl (Merck), pH 7.5	50 mM	(18)
	Dithiothreitol (DTT) (Sigma-Aldrich)	0.1 mM	
	Lysozyme (Sigma-Aldrich)	1 mg/ml	
2	Tris-HCl, pH 7.5	50 mM	(this article)
	Glycerol (Merck)	5% v/v	
	Triton X-100 (Sigma)	0.1%	
	Protease Inhibitor Cocktail Set IV (Calbiochem, Darmstadt, Germany)	5 μl/ml	
3	Tris-HCl, pH 7.5	20 mM	(Novagen pET system manual)
	NaCl (Merck)	500 mM	
	Lysozyme	0.1 mg/ml	
5	NaH_2_PO_4_ (Merck)	50 mM	
	NaCl	300mM	(17)
	Lysozyme	1 mg/ml	
	Protease Inhibitor Cocktail Set IV	5 μl/ml	
	Triton X-100	1% v/v	
	DNase (Invitrogen)	5 μg/ml	
4	NaH_2_PO_4_	20 mM	
	NaCl	500 mM	(this article)
	Urea	6 M	

### SDS–PAGE and western blot analysis.

The pellets from 1 ml samples were suspended in 50 μl phosphate-buffered saline (PBS, pH 7.4) (Sigma-Aldrich), mixed with 50μl 2X Laemmli Sample Buffer (Bio-Rad, CA, USA) plus 5% (v/v) β-mercaptoethanol (Sigma-Aldrich) and were boiled for 10 min. The resulting cocktail was centrifuged at 13000 g for 30 min at 4°C, and 30 μl of the soluble fraction from the supernatant was loaded onto a 8–16% polyacrylamide Amersham ECL gel (GE Healthcare, Freiburg, Germany) and run for 1 h at 160 V using a Amersham ECL Gel box (GE Healthcare) and 1X Tris-Glycine-SDS (Sigma-Aldrich) as running buffer. The gels were stained with 0.2% Coomassie Brilliant Blue R-250 (Sigma–Aldrich) for 1 h and destained with 5% methanol, 7.5% acetic acid (Sigma-Aldrich).

For western blots, the proteins were transferred onto PVDF membranes (Millipore, Bedford, USA) in a Semi-Dry Transfer Cell (Bio-Rad Laboratories Inc., CA, USA) at 90 V for 1 h in 1X Tris-Glycine buffer with 10% methanol (Sigma-Aldrich). Membranes were blocked with Tris-buffered saline (TBS)–skim milk (5%)-Tween 20 (0.05%) (Sigma-Aldrich) for 1 h at room temperature. Murine IgG1 monoclonal anti-penta His antibody (Thermo Fisher Scientific, Waltham, MA, Cat No. 34660) was diluted 1:2000 in the blocking buffer, applied to the membrane and incubated for 1 h at room temperature. Membranes were washed three times with TBS−0.05% Tween 20 for 5 min each and goat anti-mouse IgG horseradish peroxidase (HRP)-conjugated secondary antibodies (Santa Cruz Biotechnology Inc., Santa Cruz, CA, USA) was then applied to the membrane at a 1:5000 dilution in the blocking buffer and incubated for 1 h at room temperature. Membranes were washed as described above and the bands were revealed with Western Blue Stabilized Substrate for Alkaline Phosphatase (Promega, USA). Multi-Tag-Marker (Roche, Mannheim, Germany) was used as positive control for western blot analysis. Bands were visualized using ImageQuant^™^ LAS 500 (GE Healthcare Life Sciences, Piscataway, NJ, USA).

For factorial design experiments, 10 μl samples were loaded onto 12% SDS polyacrylamide gels and the proteins were separated at 150 V for 160 min in an electrophoresis unit (SE300 mini VE, Hoefer, Holliston, MA, USA) (19). The gels were then stained with Coomassie Blue as described above. The experiments were repeated twice at four weeks interval. Quantification of the bands corresponding to recombinant Rv1733c in SDS-PAGE gels was done using ImageQuant TL software (GE Healthcare Life Sciences) after background subtraction.

For western blotting using patient’s serum, the membrane was incubated with the serum from a consented tuberculosis positive case (1:50 dilution in TBS) for 2 h at room temperature, followed by the addition of HRP-conjugated rabbit anti-human IgG (1:5000 in TBS) (Dako, Glostrup, Denmark) for 2 h at room temperature. The protein band was then developed using diaminobenzidine (0.1% H _2_ O _2_ + 10 mg DAB in 10 ml of TBS).

### Blue native polyacrylamide gel electrophoresis (BN-PAGE).

BN-PAGE was performed in 4–16% Bis-Tris gel (NativePAGE ^™^ Novex, Invitrogen) according to the manufacturer’s recomendations with a few modifications. Briefly, cells suspended in PBS were lysed by sonication and the supernatant was mixed with 4X Native PAGE Sample Buffer (Invitrogen) supplemented with Native PAGE 5% G-250 Sample Additive. Dithiothreitol (DTT) (Sigma-Aldrich) was added in a final concentration of 1.25% v/v into every the other sample and 20 μl of each sample was loaded onto the gel. The electrophoresis was carried out using light cathode buffer (NativePAGE ^™^ Running Buffer with 0.002% Coomassie G-250) in the cathode tank and NativePAGE ^™^ Running Buffer as the anode buffer until the Coomassie front reached the end of the gel at a constant 150 V. Gel staining and western blotting were carried out as mentioned above.

### Large scale production of recombinant Rv1733c.

For production of milligram quantities of the recombinant protein, optimized conditions were scaled up accordingly. The *E. coli* BL21 harboring pET23a/*rv1733* construct was grown in 200 ml of Terrific-Broth in 1 L baffled flask at 37°C to an OD _600_ of 0.6, followed by induction with 0.4 mM IPTG for 10 h. The cells were then pelleted by centrifugation and stored at −20°C before being lysed.

The cell pellets were resuspended in the selected lysis buffer (20 ml/g cell pellet, 20 mM NaH _2_ PO _4_ , 500 mM NaCl and 6 M urea, pH 8) (Merck, Darmstadt, Germany) and incubated for 15 min on ice. The suspension was sonicated six times for 20 s and cell debris was removed by centrifugation (13000 g, 30 min, 4°C). The supernatant was passed through 0.45 μm filter before loading on complete His-Tag Purification Column (Roche, Mannheim, Germany). The column was washed extensively with washing buffer (20 mM NaH _2_ PO _4_ , 500 mM NaCl, pH 8) until the OD _280_ of the buffer reached below 0.01. The proteins were then eluted using a column volume of 500 mM imidazol (Sigma-Aldrich), pH 8 containing complete protease inhibitor (Sigma-Aldrich). The eluted fractions were combined and dialyzed against PBS (pH 7.4), and quantified by Bradford total protein assay kit (ZellBio GmbH, Ulm, Germany). LPS contamination was evaluated by the Limulus amoebocyte lysate (LAL) assay (Cambre, USA).

### Statistical analyses.

ANOVA (SPSS 16.0 for Windows, SPSS, Chicago, USA) was used to determine the significance of each factor on recombinant protein expression. To assess the strength of correlations between the variables and statistical significance, separate bivariate analyses were performed by use of the non-parametric Spearman’s rank correlation test.

## RESULTS

**Fig. 2.**
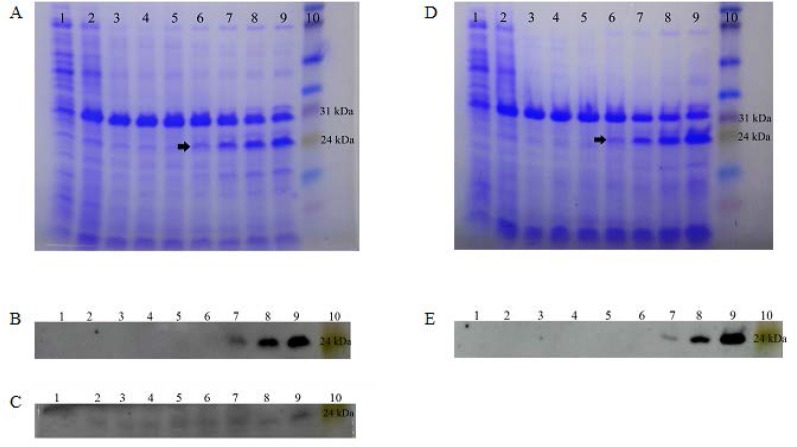
SDS-PAGE gels stained with Coomassie Blue and anti-polyhistidine western blot analyses of recombinant Rv1733c expressed in *E. coli* BL21 (DE3). A and B: supernatants after induction with 0.3 mM IPTG. C: cell pellets after induction with 0.3 mM IPTG. D and E: supernatants after induction with 0.4 mM IPTG. lane 1: time 0 (non-induced), lane 2: Time = 1 h, lane 3: T = 2 h, lane 4: T = 3 h, lane 5: T = 4 h, lane 6: T = 5 h, lane 7: T = 6 h, lane 8: T = 7 h, lane 9: T = 8 and lane 10: full-range Rainbow molecular weight marker. The protein band is indicated with arrow.

**Fig. 3.**
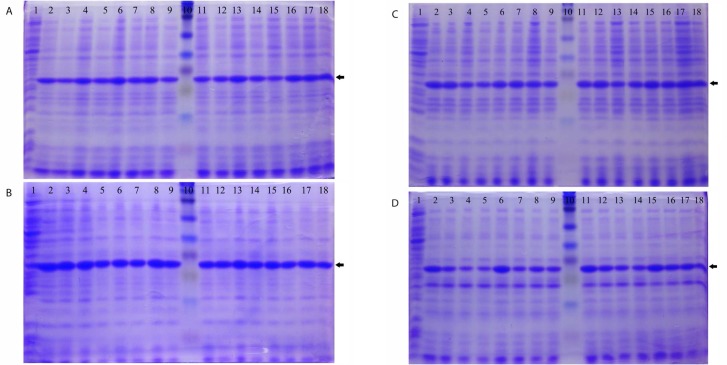
SDS-PAGE gels stained with Coomassie Blue for recombinant Rv1733c expressed in *E. coli* BL21 (DE3) in different media, IPTG concentrations and time points. A: 2x Yeast extract and Tryptone broth was used as culture medium; B: Terrific Broth medium; C: Super Broth medium; D: Luria-Bertani broth with 0.5% NaCl. lane 1: time 0 (non-induced), lane 2: IPTG = 0.1 mM & Time = 7 h, lane 3: IPTG = 0.1 mM & Time = 8 h, lane 4: IPTG = 0.1 mM & Time = 9 h, lane 5: IPTG = 0.1 mM & Time = 10 h, lane 6: IPTG = 0.2 mM & Time = 7 h, lane 7: IPTG = 0.2 mM & Time = 8 h, lane 8: IPTG = 0.2 mM & Time = 9 h, lane 9: IPTG = 0.2 mM & Time = 10 h, lane 10: full-range Rainbow molecular weight marker, lane 11: IPTG = 0.3 mM & Time = 7 h, lane 12: IPTG = 0.3 mM & Time = 8 h, lane 13: IPTG = 0.3 mM & Time = 9 h, lane 14: IPTG = 0.3 mM & Time = 10 h, lane 15: IPTG = 0.4 mM & Time = 7 h, lane 16: IPTG = 0.4 mM & Time = 8 h, lane 17: IPTG = 0.4 mM & Time = 9 h, lane 18: IPTG = 0.4 mM & Time = 10 h. The protein band is indicated with arrow.

**Fig. 4.**
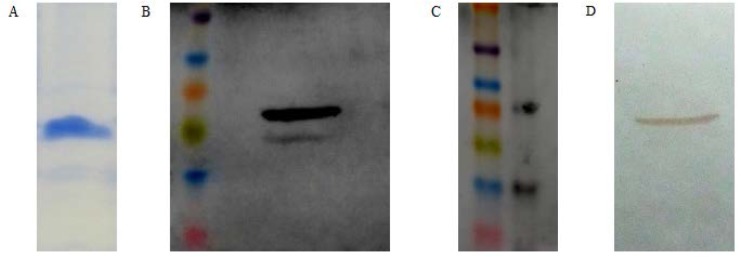
Recombinant Rv1733c purified using polyhistidine (His)-tag affinity column chromatography. A: SDS-PAGE gel stained with Coomassie Blue. B: Western blot analysis using an anti-His antibody. C: Positive control-Multi-Tag-Marker (Roche) for anti-His western blotting. D: Western blot analysis using a tuberculosis patient’s serum.

The *rv1733c* sequence was successfully cloned into pET23a plasmid in *E. coli* BL21. Preliminary induction experiments with 0.3 and 0.4 mM IPTG at 37°C showed that the expression of soluble His-tagged Rv1733c occurred 6 to 8 h after induction in the cytoplasm of *E. coli* BL21 (Fig. 2). Lower induction temperatures (30°C and 25°C) caused a marked decrease in Rv1733c expression (data not shown). Results from the factorial design of experiments (Fig. 3) showed that media composition had the most significant effect on the expression of soluble recombinant Rv1733c (P = 0.000), while IPTG concentration and duration of induction did not have a significant influence (Table 2). No significant correletion was found between the variables. The highest expression of the recombinant protein occurred with 0.4 mM of IPTG at 37°C in Terrific Broth after 10 h. The results obtained from the factorial experiments were confirmed by the orthogonal array design of experiments. Lysis buffer 5 most efficiently released the recombinat protein from cell pellets and the result of BN-PAGE confirmed the need for a denaturing buffer for purification; recombinant Rv1733c band was observed in the stained BN-PAGE gel but the Histag was not exposed in native condition resulted in the lack of protein detection in its western blot either in the presence or absence of DTT (data not shown). The recombinant Rv1733c detected with anti-His antibody and serum from the tuberculosis patient (Fig. 4). It was successfully purified with a yield of 0.5 g/L of TB medium and the endotoxin content was below 50 IU/mg of the recombinant protein.

## DISCUSSION

Comprehensive data mining and bioinformatic analyses have identified Rv1733c as the 14th most promising candidate among 189 putative vaccine candidates from the entire genome of *M. tuberculosis* (20). Our bioinformatics predictions characterized Rv1733c as a pertinent candidate for vaccine against tuberculosis as was also reported in a recent bioinformatic study (21). These bioinformatic predictions have been endorsed by *in vitro* studies which reported that Rv1733c is preferentially recognized by T-cells from latently *M. tuberculosis*-infected individuals, who are able to control the infection in the long run and remain healthy (22–24). This suggests that the immune response to Rv1733c may play a protective role against tuberculosis and that this antigen has the potential to be an effective tuberculosis vaccine (4).

An important factor affecting recombinant protein expression and/or solubility is post-induction temperature (14–15). Reduced temperatures have been shown to be effective in improving the solubility of a number of difficult-to-express proteins and would lead to a lower probability of plasmid loss (12, 14, 15). On the other hand, higher temperature (37°C) allows for more biomass and a larger protein quantities to be produced per litre of culture medium (14). We also found that the best expression of the recombinant Rv1733c in its soluble form occurred at 37°C.

Factorial design of experiments has been used for optimization of a number of induction conditions to provide information on interactions between variables (15). Our results using the factorial design of experiments presented no significant correlation between the variables suggesting that each effect should be evaluated independently. These observations were also confirmed by the orthogonal array design (data not reported).

Broedel et al. reported that optimization of medium composition improved the expression of recombinant proteins in *E. coli* (25). In contrast, Vincentelli and colleagues compared SB medium, 2x YT broth and TB medium, and showed that culture media composition was not a major determinant in the expression of soluble forms of recombinant proteins in both prokaryotes and eukaryots (14). Our observation agrees with Broedel’s suggestion as the TB medium worked far better (P = 0.000) than 2x YT, SB and LB media in expression of recombinant Rv1733c by *E. coli* BL21. TB is a rich medium with a high concentration of yeast extract, has a better buffering capacity compared to the other media and contains glycerol which has an stabilizing effect on proteins (26). Concentration of the inducer and the length of induction period also affect recombinant protein yields (27). We found that 0.4 mM IPTG, the maximum level recommended by Novagen pET system manual, significantly enhanced the amount of recombinant Rv1733c after 7 to 10 h.

It has been reported that the nature of the lysis buffer can strongly influence the partitioning of a target protein into the soluble or insoluble fraction (16). Proteins containing membrane-associated domains may partition into the soluble fraction by addition of appropriate detergents to the lysis buffer. However, the addition of detergent may affect downstream purification procedures [Novagen pET system manual]. Among the different lysis buffers employed, the denaturing lysis buffer containing 6 M urea protected the recombinant protein from proteases and exposed the polyhistidine tag required for purification. BN-PAGE result also showed that in non-denaturing condition, the His-tag of recombinant Rv1733c is not exposed regardless of reducing or non-reducing conditions. Hence, the protein was successfully purified and eluted from the His-tag column in its native conformation and was detected by the serum from a tuberculosis patient in western blot analysis.

In conclusion, we believe that this research is the first report approaching the optimization of recombinant Rv1733c expression in *E. coli*. We believe that the purified Rv1733c recombinant protein from *M. tuberculosis* might be a good candidate for vaccine production against tuberculosis. Further studies are needed to evaluate the stability and *M. tuberculosis*-specific immune responses towards Rv1733c protein *in vivo.*
